# Who gets vaccinated in a measles-rubella campaign in Nepal?: results from a post-campaign coverage survey

**DOI:** 10.1186/s12889-021-12475-0

**Published:** 2022-02-03

**Authors:** M. Carolina Danovaro-Holliday, Dale A. Rhoda, Mona Lacoul, Mary L. Prier, Jhalak Sharma Gautam, Tara Nath Pokhrel, Sameer Mani Dixit, Rajesh Man Rajbhandari, Anindya Sekhar Bose

**Affiliations:** 1 Department of Immunization, Vaccines and Biologicals (IVB), World Health Organization (WHO), 20, Ave Appia, 1211 Geneva, Switzerland; 2 Biostat Global Consulting, Worthington, OH USA; 3 Country Office Nepal, WHO, Kathmandu, Nepal; 4 Family Welfare Division, Department of Health Services, Government of Nepal, Kathmandu, Nepal; 5 Center for Molecular Dynamics (CMDN), Kathmandu, Nepal

**Keywords:** Immunization, Measles-rubella, Vaccination campaign, Nepal, Vaccination coverage survey

## Abstract

**Background:**

Following the 2015 earthquake, a measles-rubella (MR) supplementary immunization activity (SIA), in four phases, was implemented in Nepal in 2015–2016. A post-campaign coverage survey (PCCS) was then conducted in 2017 to assess SIA performance and explore factors that were associated with vaccine uptake.

**Methods:**

A household survey using stratified multi-stage probability sampling was conducted to assess coverage for a MR dose in the 2015–2016 SIA in Nepal. Logistic regression was then used to identify factors related to vaccine uptake.

**Results:**

Eleven thousand two hundred fifty-three households, with 4870 eligible children provided information on vaccination during the 2015–2016 MR SIA. Overall coverage of measles-rubella vaccine was 84.7% (95% CI: 82.0–87.0), but varied between 77.5% (95% CI: 72.0, 82.2) in phase-3, of 21 districts vaccinated in Feb-Mar 2016, to 97.7% (CI: 95.4, 98.9) in phase-4, of the last seven mountainous districts vaccinated in Mar-Apr 2016. Coverage in rural areas was higher at 85.6% (CI: 81.9, 88.8) than in urban areas at 79.0% (CI: 75.5, 82.1). Of the 4223 children whose caregivers knew about the SIA, 96.5% received the MR dose and of the 647 children whose caregivers had not heard about the campaign, only 1.8% received the MR dose.

**Conclusions:**

The coverage in the 2015–2016 MR SIA in Nepal varied by geographical region with rural areas achieving higher coverage than urban areas. The single most important predictor of vaccination was the caregiver being informed in advance about the vaccination campaign. Enhanced efforts on social mobilization for vaccination have been used in Nepal since this survey, notably for the most recent 2020 MR campaign.

**Supplementary Information:**

The online version contains supplementary material available at 10.1186/s12889-021-12475-0.

## Introduction

In Nepal, the Expanded Program on Immunization (EPI) was launched in 1977 and was expanded to all district by 1989. Initially, the national immunization schedule included one dose of measles vaccine recommended at 9 months [1, 2]. Since conducting a measles catch-up campaign in 2004–2005 and a follow-up campaign in 2008, Nepal saw a 98% drop in reported measles cases, from 12,074 in 2004 to 190 in 2010 [2]. In the pre-rubella vaccine era in Nepal, up to 1426 children or 192 cases per 100,000 live births were estimated to be born each year with congenital rubella syndrome (CRS). In 2013, the measles-rubella (MR) vaccine was introduced in routine immunization following an MR vaccination campaign [1, 3].

To maintain the achievements in measles and rubella control and to prevent a measles outbreak after the devastating earthquake of 2015, a MR (and polio) supplementary immunization activity (SIA), or “MR campaign”, was conducted in four phases covering all districts between 2015 and 2016. The campaign targeted children aged 6 months to 5 years in earthquake affected districts and 9 months to 5 years in other districts. MR campaign phases were:

- First phase conducted in in Aug-Sep 2015, in the 14 districts highly affected by the 2015 earthquake.
  - ◦ This phase was followed by the introduction of a second MR dose in the national routine immunization schedule targeting children aged 15 months
- Second phase in 33 districts Feb-Mar 2016,
- Third phase in 21 districts starting later in Feb-Mar 2016, and
- Fourth phase in the last seven mountainous districts Mar-Apr 2016.

The overall administrative coverage of the 2015–2016 campaign was above 95% [4]. The planning for each SIA phase was done at national and district levels. Nepal has very well spread out network of Female Community Health Volunteers (FCHV) who were utilized for community mobilization. Opinion leaders at national as well as sub-national levels are always involved in major public health activities like an SIA and they also helped in communication and social mobilization. For immunization, Nepal has an institutionalized mechanism of community ownership and engagement of opinion leaders through Immunization Coordination Committees at successive subnational levels; members include elected representatives (Mayors, Chairpersons of municipalities, municipal ward members etc.) and members of civil society organizations. Health workers and independent monitors were deployed for house to house visit to identify missed children and to vaccinate through campaign. Key elements added to SIA planning for the SIA in earthquake-affected areas included: a) microplanning to accommodate displaced populations - additional session sites were planned in makeshift camps where affected population were residing along with usual one site per ward (standard practice for campaign planning); b) revamping of cold chain - Identification of cold-chain equipment destroyed, replaced with passive cold -chain equipment for vaccine storage; and c) In places where vaccine transportation was an added challenge after the earthquake, alternate arrangements were made with porters.^1^

Since 2017, MR1 coverage in routine immunization in Nepal has remained at or above 90%, while MR2 coverage has gradually increased from 59% in 2017 to an estimated 76% in 2019 at the national level [2]. And yet, measles and rubella cases have continued to occur. Coverage with two doses of measles containing vaccine (MCV) has not reached 95% and latest surveys have shown geographic inequities between provinces [5]. Another vaccination campaign was planned for 2019, but it was postponed to 2020, and this last MR, plus polio, campaign was conducted in the midst of the COVID-19 pandemic [6].

A household survey was done in 2017 to assess coverage achieved in the 2015–2016 post earthquake measles-rubella immunization campaign. Here we report campaign coverage and explore correlation of some socio-epidemiological parameters and programmatic interventions with vaccination uptake in the campaign. We also discuss how these survey results vindicate the strategies undertaken by the Nepal national immunization program (NIP) for the MR-polio vaccine campaign conducted in Nepal in 2020.

## Methods

### 2017 vaccination coverage survey

The MR post-campaign coverage survey (PCCS) was conducted with the main objective of estimating coverage obtained by the MR vaccination campaign 2015–2016 by rural/urban setting for each phase of the SIA campaign in the target age group of children. A sub-objective was to assess covariates positively associated with SIA dose uptake.

At the time of the MR campaign, Nepal was in the phase of transformation from unitary governance to federal government. Nepal did not have a federal governance structure and the country was administratively divided into five Development Regions and 75 districts with no provinces. Nepal transitioned to a federal governance structure fully in 2018 with seven provinces and 753 municipalities. In our analysis, we present weighted estimates of immunization coverage by phase (as per a-priori sampling design) as well as by province (by re-grouping the districts by province).

Table 1 shows correspondence between the phases of the MR SIA campaign and present provincial structure. Each district was subdivided into Village Development Committees (VDC) and Urban municipalities and all VDCs and municipalities in a district conducted the campaign at the time periods shown.

**Table 1:** Measles-Rubella vaccination campaign 2015–2016 – districts covered, age-group and target population, campaign duration and administrative coverage

Campaign Phase	Number of Districts^**a**^	Corresponding province (Number of districts, Full/ Partial)	Earthquake affected	Target age group for MR-SIA	Duration of campaign	Target population	Administrative Coverage^**c**^ (%)
1	14	Bagmati (12,P), Gandaki (1,P), Province-1 (1,P)	Y	6–59 months	15 Aug - 15 Sept 2015	500,344	91%
2	33	Gandaki (8,P), Lumbini (11,P), Karnali (5,P), Sudurpaschim (9,P)	N	9–59 months	7 Feb - 6 Mar 2016	1,090,504	103%
3	21	Province 1 (12,P), Province 2 (8, F) Bagmati (1,P)	N	9–59 months	21 Feb - 21 Mar 2016	1,371,798	99%
4^b^	7	Province-1 (1,P) Gandaki (2,P) Karnali (4,P)	N	9–59 months	15 March - 12 Apr 2016	39,617	117%
National	75	7 provinces (75 districts)	–	–	–	3,002,263	99%

^a^At the time of campaign there were 75 districts in Nepal. Later two districts were split taking the number of districts to 77

^b^Mountain districts

^c^This is obtained by dividing the number of doses administered divided by the estimated target population from 2011 census projections; limitations of admin coverage include target population inaccuracies, older children sometimes vaccinated and unquantifiable mismatch between where people reside and where they get vaccinated, especially in urban areas

### Study population and sampling

The sampling universe included all children aged 9–59 months at the time of the 2015–2016 campaign (6–59 months in earthquake affected districts). An overlapping subset of children aged 12–23 months at the time of the survey was studied for routine immunization (RI) coverage as well as women who had given birth in the previous 12 months to estimate tetanus protection at birth; these two populations and survey results of these subsets are not described here.

The PCCS was conducted following the World Health Organization (WHO) vaccination coverage cluster survey reference manual (2015 working draft; finalized in 2018 and revised (minimally) in 2019 and again (minimally) in 2021) [7]. For variables related to religion and caste/ethnicity, we follow the definitions used by Government of Nepal programs and those used in the latest Nepal Demographic and Health Survey (NDHS). The country was divided into seven strata, by phase of campaign and by rural (VDC) and urban (municipality) setting: the first three phases of the campaign had both urban and rural settings and formed six strata; the fourth phase included only rural settings (VDC) adding the seventh stratum to the survey. Urban and rural estimates were combined using a weighted average to estimate coverage by campaign phase.

The primary sampling units (PSU) sampling frame was the list of enumeration areas (EAs) with population and household information from VDC or municipal setting developed by the Central Bureau of Statistics from the 2011 Population Census. EAs that were determined a priori to be of insufficient size (mostly in rural areas) were merged with a nearby PSU and those determined to be too large (mostly in urban areas) were segmented into 250–300 households in each segment and one segment was randomly selected. Selection of PSUs was done using probability proportional to the number of households in the EA in the 2011 census. A household listing and mapping exercise was done in each selected PSU right before the survey. Households were selected using a sampling interval supported by a digital tool built into the tablets used for data collection.

### Sample size estimation

Sample size was calculated using the method given in Annex B of the 2015 draft WHO coverage survey manual [8]. The sample size for each stratum was calculated for the campaign outcome assuming vaccination coverage of 95%, a desired precision no wider than ±5% for the two-sided 95% confidence interval which gave the effective sample size as 162. The design effect was assumed to be no greater than 2.5 (estimated intra-cluster coefficient (ICC) of 1/6 and 10 respondents per cluster) and so sample size per stratum would be 410 (rounded) with a target of 10 children to be selected in each of 41 clusters. Applying birth and infant mortality rates, an average of 4 and 3 households would need to be visited to find an eligible child for MR campaign in urban and rural strata, respectively. Non-response was assumed to be 10%. Thus, the sample per stratum included 41 clusters, with an objective to visit 44 and 35 households per cluster in urban and rural areas, respectively. All eligible children in a selected household were included in the survey if the caregiver consented. Up to three visits were attempted per selected household to secure a completed interview.

### Data collection

Field work took place from February to June 2017 for six strata and was deferred to October 2017 for the last stratum because of national elections. WHO Nepal engaged an external agency, Center for Molecular Dynamics (CMDN) for survey data collection. All enumerators and supervisors were trained through a 5-day training session that included practical work and pilot testing of the tools, and a 1-day refresher session in October for the last strata. Training sessions were led by experts from Department of Health Services and WHO. Data was collected using tablets and standardized questionnaires developed using CSPro [9]. MR-SIA campaign dates for different phases were pre-inputted in the program, so that the data collection tools would automatically identify if a sampled child was in the eligible age-group for that district at the time of the campaign. The interview included questions on campaign vaccination status and reasons for missing vaccination. Campaign cards were not used so only caregiver recall could be used to ascertain MR vaccination during the campaign. The questionnaires were developed in English and then translated into Nepali language (and back translated into English to verify accuracy). Pre-testing was carried out in a non-sample location in Kathmandu valley, which includes both urban and rural areas. The final questionnaire was bilingual on paper (English and Nepali), but the questions were asked in Nepali or the local language. The surveyor teams included local enumerators who could fluently speak the local language, and who could translate questions in the field. This was the case for all local level data collection activities. Additionally, all interviews were recorded on digital recorders to ensure no information was left out while back translating. Each tablet was synchronized and backed up daily to ensure safety of the data. To ensure adherence to the survey protocol and maximize data quality, field monitoring activities were done regularly by staff from CMDN, WHO and staff from National Immunization Program (NIP).

### Ethical considerations

Ethical approval was obtained from Nepal Health Research Council (NHRC) prior to start of the survey (Reg. no. 33O/2O16). All the participants were informed about the survey purpose and recruitment procedures and about their right to decide whether to participate in the survey and to abstain from answering particular question/s. Informed consent was sought before starting the interview. Data was maintained safe and the database was deidentified. See section on “Ethics approval and consent to participate” for further details.

### Statistical analysis

Survey weights were calculated in two steps: base weights were calculated as the inverse of the cumulative probability of selection over four stages (enumeration area, segment, household, child). Then base weights were post-stratified so the sum of weights in each stratum would match administrative province-level estimates of 2014/15 population under age 5 years [7].

Data was cleaned using SPSS and exported into Stata. Descriptive household demographics analyses and reasons for missing SIA vaccination are presented as weighted proportions (unless otherwise mentioned) and measures of central tendencies for continuous variables. The WHO survey analysis package Vaccination Coverage Quality Indicators (VCQI) was used to automate the calculation of coverage-related indicators [10, 11]. Weighted coverage estimates and 95% confidence intervals (CI) using the Wilson method were calculated for each stratum and the country, taking into account the sampling design [12]. Rao-Scott chi-square tests were used to compare coverage between various subgroups [13]. A *p*-value < 0.05 was considered statistically significant. Logistic regression was employed to assess demographic and program intervention covariates for association with whether a child received the campaign dose (response variable).

In an early screening step, numerous explanatory variables were assessed with univariable logistic regression. For each such regression, five performance metrics were noted:

1. The *p*-value of the regression: any covariate with a p-value smaller than 0.25 was considered a candidate to enter a later multivariable logistic regression.
2. The concordance statistic (c-statistic): The proportion of all possible pairs of vaccinated and unvaccinated children where the explanatory variable correctly predicted the response variable (vaccination status). A perfect model would yield a value of 1.0, meaning that every child who was vaccinated would be assigned a higher probability of the vaccination outcome than every child who was not vaccinated. When the value falls between 0 and 1.0, it represents the portion of pairs of vaccinated and unvaccinated children for whom the predicted probability is ordered correctly; a c-statistic of 90% is considered ‘outstanding discrimination’. This metric is also known as the area under the receiver operating characteristic (ROC) curve [14].
3. The proportion of children for whom the model predicts the probability of vaccination is > 50%.
4. The proportion of vaccinated children for whom the model predicts probability of vaccination is > 50%. Ideally 100%.
5. The proportion of un-vaccinated children for whom the model predicts probability of vaccination is < 50%. Ideally 100%.

A multivariable model was constructed using purposeful variable selection, starting with all variables in the model whose univariable *p*-value was smaller than 0.25 and then removing, one-by-one, the covariate with the highest p-value over 0.05, unless removing that variable changed the coefficients on one or more of the remaining covariates by more than 20% [15]. When that process was finished, the so-called main effects multivariable logistic regression model was estimated, and its utility was evaluated using the same five criteria used for univariable models. Interactions were not explored because there were an abundance of two-way interaction possibilities and none had been identified a priori as being of programmatic interest.

Regression analyses were conducted with Stata version 16 using syntax that takes the complex sample design into account [16]. The main effects model was assessed for goodness of fit to the data using the un-trimmed survey weights and three modified sets of weights that were trimmed using modest, medium, and aggressive trimming criteria; those methods and results are described in the electronic supplement [17, 18].

## Results

A total of 11,275 households were sampled and 11,253 were interviewed for a response rate of 99.8%. In the interviewed households, 4870 children were identified as eligible for a MR dose in the 2015–2016 campaign and interviews were completed for all (Table 2).

**Table 2:** Number of households, number of interviews and response rates according to residence (unweighted), Nepal PCCS, 2017

	Rural	Urban	Total
**Household interviews**			
Sampled households (N)	5740	5535	11,275
Household interviewed (N)	5740	5513	11,253
Household response rate^a^ (%)	100	99.6	99.8
**Children for MR in campaign**			
Eligible (N)	2640	2230	4870
Interviewed (N)	2640	2230	4870
Response rate^b^ (%)	100	100	100
Overall response rate^c^ (%)	100	99.6	99.8

^a^Household response rate = 100 x Household interviewed/Sampled household

^b^Response rate = 100 x Interviewed/Eligible

^c^Overall response rate = Product of household response and child level response rate

### Household characteristics (unweighted proportions)

Mean household size was 4.2 (SD: ±1.6). Among the 11,253 households interviewed, 4163 (37.0%) had at least one child eligible for the 2015–2016 MR vaccination campaign. Almost a third (31.0%) of the persons in the households interviewed were aged < 15 years. Almost all households had electricity (including solar electricity) (98.6%), access to improved source of drinking water (97.1%), an improved toilet facility (93.8%) and access to a mobile phone (98.4%). Nearly half of the respondents (45.4%) had migrated to their current location, (urban 54.8%; rural 12.3%). Among those who migrated, most had been living in the place of migration for more than 5 years in both the urban (70.9%) and rural settings (79.8%); 27.2% of households had at least one family member working abroad (usually in India).

### Vaccination coverage during the 2015–2016 MR campaign

Overall, 84.7% (95% CI: 82.0–87.0) of eligible children received MR vaccine during the campaign. However, MR coverage varied from 77.5% (95% CI: 72.0, 82.2) in phase-3 to 97.7% (CI: 95.4, 98.9) in phase-4. The coverage in rural areas was higher at 85.6% (CI: 81.9, 88.8) than in urban areas at 79.0% (CI: 75.5, 82.1) (Table 3) Survey coverage was lower than that estimated through administrative reporting of number of doses administered divided by estimated target population in all strata (Table 1).

**Table 3:** National Measles-Rubella Campaign Coverage by selected characteristics, Vaccination Coverage Survey, Nepal, 2017

	Vaccinated during campaign (%)	95% CI (%)	Design effect	N	Weighted N
**Nepal**	**84.7**	**(81.5, 87.4)**	**8.6**	**4870**	**2,959,179**
**Province**					
Province 1	87.9	(83.5, 91.2)	2.9	806	494,326
Province 2	70.0	(61.3, 77.5)	5.9	730	613,411
Province 3 (Bagmati)	81.1	(73.3, 87.0)	9.3	1163	629,560
Province 4 (Gandaki)	87.4	(76.1, 93.8)	8.9	495	254,047
Province 5 (Lumbini)	91.4	(86.7, 94.5)	2.9	616	503,233
Province 6 (Karnali)	98.0	(93.9, 99.4)	5.6	740	177,351
Province 7 (Sudur Paschim)	96.0	(90.1, 98.4)	3.1	320	287,251
**SIA phase**					
Phase-1	82.6	(78.6, 85.9)	3.2	1099	586,721
Phase-2	92.6	(89.9, 94.6)	5.4	1521	1,139,229
Phase-3	77.5	(72.0, 82.2)	10.6	1528	1,180,280
Phase-4	97.7	(95.4, 98.9)	0.4	722	52,949
**Urban/Rural**					
Rural	85.6	(81.9, 88.8)	6.6	2640	2,530,810
Urban	79.0	(75.5, 82.1)	3.8	2230	428,369
**Earthquake Affected District**					
Yes	82.6	(77.8, 86.5)	3.7	1100	587,267
No	85.2	(81.3, 88.4)	9.8	3770	2,371,912
**Caregiver Education**					
Never Schooling/Illiterate	84.3	(75.5, 90.3)	3.3	312	266,692
Literate but no Formal Education	94.1	(89.9, 96.6)	1.1	212	192,688
Less than one Class	74.1	(64.1, 82.0)	9.0	809	484,774
Primary	88.3	(82.7, 92.3)	5.1	888	601,649
Secondary	86.9	(82.5, 90.2)	4.2	1261	756,574
School Leaving Certificate and above	84.0	(80.1, 87.2)	3.2	1298	611,961
Don’t Know	84.9	(70.9, 92.8)	2.2	90	44,840
**Caregiver Marital Status**					
Married	84.6	(81.3, 87.4)	8.7	4746	2,895,285
Not married	97.5	(92.0, 99.3)	0.3	33	17,435
Missing	85.4	(71.6, 93.1)	2.2	91	46,459
**Religion**					
Hindu	84.6	(81.2, 87.6)	8.5	4195	2,530,329
Buddhist	91.3	(85.2, 95.0)	2.4	320	209,307
Muslim	76.2	(64.8, 84.7)	3.5	239	142,576
Not Hindu/Buddhist/Muslim	83.5	(64.5, 93.4)	4.7	116	76,966
**Caste/Ethnicity**					
Brahmin/Chhetri	89.9	(86.8, 92.3)	4.0	1854	888,628
Other Terai Caste	64.9	(53.3, 75.0)	6.3	451	319,737
Dalit	83.3	(76.3, 88.5)	4.1	596	432,476
Janjatis	87.7	(82.9, 91.2)	6.7	1602	1,081,623
Muslim	76.0	(60.3, 86.8)	6.2	236	145,195
Others	88.5	(78.6, 94.2)	1.9	131	91,520
**Income Source**					
Professional \Technical\Managerial	79.5	(63.5, 89.6)	2.8	101	23,394
Jobber/Service holder	85.1	(79.8, 89.2)	4.0	892	411,813
Sales and services	85.5	(79.6, 89.9)	5.0	914	344,431
Skilled manual	74.8	(67.3, 81.1)	5.2	787	472,115
Unskilled manual	80.5	(71.9, 87.0)	2.9	306	244,702
Agriculture	89.1	(85.5, 91.9)	4.4	1651	1,338,024
Others	80.4	(70.1, 87.8)	2.7	209	121,107
**Migration**					
Didn’t migrate	84.9	(81.3, 87.9)	8.6	3981	2,633,395
Migrated < 12 months ago	75.2	(52.4, 89.3)	2.6	49	14,929
Migrated 1–5 years ago	83.2	(78.4, 87.0)	2.9	840	310,855
**Someone in the Family Works Abroad**					
Yes	87.4	(82.6, 91.0)	5.5	1322	880,154
No	83.5	(79.6, 86.8)	8.6	3548	2,079,026
**Ecological Belt**					
Mountain	96.1	(89.3, 98.6)	11.0	885	222,569
Hill	88.3	(85.3, 90.8)	3.6	1923	1,229,944
Terai (Plain)	80.0	(74.4, 84.7)	9.0	2062	1,506,666

Of all caregivers (4870), 83.3% (95% CI: 79.8–86.3) reported having been informed about the campaign, with a higher proportion informed in rural (87.8%; 95% CI: 84.1–90.8) compared to urban areas (81.6%; 95% CI: 77.4–85.2) (Table 3).

Of 4223 caregivers who also stated the source of this prior information, 64.2% identified the female community health volunteer (FCHV) as the source. The second most common source of information was local health workers (LHW) at 21.4%. For rural residents, FCHV or LHW were the primary sources of prior information for 82.2 and 16.8% caregivers; for urban residents, these proportions were 57.2 and 23.2%. Some received the information from more than one source (Table 3).

Among the 759 (15.3%) caregivers of children not vaccinated during the campaign, the most common reason for non-vaccination was reported as lack of information by 93.1%, while 29.1% reported some form of obstacles to reach vaccination session sites and 19.9% did not feel the need to get their children vaccinated or had some fear of adverse reactions (the question allowed multiple responses).

### Correlates of receiving SIA measles rubella (MR) vaccination

Table 4 shows survey estimated campaign coverage levels for different levels of 17 demographic and program intervention variables along with the odds ratio, *p*-value, and model performance metrics of univariable regression. The first entry in the table is quite notable: of the 4223 children whose caregivers knew about the campaign, 96.5% received the MR dose and of the 647 children whose caregivers did not know about the campaign, only 1.8% received the MR dose. The odds ratio for that variable is 1477; the *p*-value is infinitesimal, and if a predicted probability of 50% were used as the threshold for predicting vaccination, the univariable regression model would predict that 87.5% of the children were vaccinated: predicting correctly for 99.7% of children who were vaccinated and for 80.1% of children who were not. While some of the other univariable predictors show some power to discriminate between children who were vaccinated and those who were not, all of those models predict > 50% probability of vaccination for every child. Of the 17 univariable models tested, the concordance statistic (c-statistic) was 90% for the variable about caregiver ‘knew of MR-SIA campaign’, 60% and above for four variables (ethnic group, source of income, province, and ecological zone), and for the remaining 12 variables, it was between 50 and 59%. The higher the c-statistic, the better the model discriminates between vaccinated and unvaccinated children [14].

**Table 4:** Logistic regression model performance of exploratory univariable models and the multivariable main effects model

Index	Variable name and categories	Got Campaign Dose % (weighted)	N (unweighted)	Unadjusted Odds Ratio	p-value (Same as univariable logistic regression ***p***-value)	Logistic regression c-stat (or area under the ROC curve)	Weighted % predicted vx’d (0.5 cut off)	Weighted % vx’d correct prediction (i.e., given they got campaign dose, what % had phat > 0.5?)	Weighted % not vx’d correctly predicted (i.e., given they didn’t get campaign dose, what % had phat < 0.5?)
1^a^	**Knew about Campaign**				**< 0.0001**	0.90	87.5	99.7	80.1
	Yes	96.5	4223	1477.21					
	No	1.8	647	Ref					
2^a^	**Urban/Rural**				**0.0087**	0.53	100	100	0
	Rural	85.6	2640	Ref					
	Urban	79.0	2230	0.63					
3	**Earthquake Area**				0.3524	0.52	100	100	0
	Yes	82.6	1100	Ref					
	No	85.2	3770	1.21					
4	**Gender**				0.8098	0.50	100	100	0
	Boy	84.5	2565	Ref					
	Girl	84.9	2305	1.03					
5^a^	**Caregiver Marital Status**				0.2009	0.50	100	100	0
	Married	84.6	4746	Ref					
	Not Married	97.5	33	7.12					
	Unknown	85.4	91	1.07					
6^a^	**Caregiver Education**				0.0717	0.55	100	100	0
	Don’t Know or less than Primary	81.2	1423	Ref					
	Primary	88.3	888	1.74					
	More than Primary	85.6	2559	1.37					
7^a^	**Caregiver Religion**				0.1012	0.53	100	100	0
	Hindu	84.6	4195	Ref					
	Buddhist	91.3	320	1.91					
	Muslim	76.2	239	0.58					
	Not Hindu/Buddhist/ Muslim	83.5	116	0.92					
8^a^	**Ethnic Group**				**< 0.0001**	0.65	100	100	0
	Hill Brahmin	87.9	579	Ref					
	Hill Chhetri	90.7	1100	1.35					
	Terai Brahmin Chettri	91.8	175	1.54					
	Other Terai Caste	64.9	451	0.25					
	Hill Dalit	88.0	360	1.01					
	Terai Dalit	79.4	236	0.53					
	Newar	84.8	295	0.77					
	Hill Janajati	85.8	899	0.84					
	Terai Janajati	91.4	408	1.46					
	Muslim	76.0	236	0.44					
	Others	88.5	131	1.06					
9^a^	**Source of Income**				**< 0.0001**	0.61	100	100	0
	Professional/Technical/Managerial	79.5	101	Ref					
	Jobber/Service holder	85.1	892	1.47					
	Sales and services	85.5	914	1.53					
	Skilled manual	74.8	787	0.77					
	Unskilled manual	80.5	306	1.07					
	Agriculture	89.1	1651	2.12					
	Others	79.8	219	1.02					
10	**Migration**				0.4184	0.51	100	100	0
	Yes	82.8	889	Ref					
	No	84.9	3981	1.17					
11	**Migration Time**				0.4706	0.51	100	100	0
	Didn’t migrate	84.9	3981	Ref					
	Migrated < 12 months ago	75.2	49	0.54					
	Migrated 1–5 years ago	83.2	840	0.88					
12^a^	**Someone in the Family Works Abroad**				0.1444	0.53	100	100	0
	Yes	87.4	1322	Ref					
	No	83.5	3548	0.73					
13^a^	**# Family Members in HH**				0.1630	0.53	100	100	0
	< =4 members in HH	86.5	2190	Ref					
	> 4 members in HH	83.5	2680	0.79					
14^a^	**# Children 9–59 months in HH**				0.2207	0.52	100	100	0
	< =1 child in HH aged 9–59 month	85.4	3503	Ref					
	> 1 child in HH aged 9–59 month	83.2	1367	0.85					
15^a^	**Child Age Group**				0.2961	0.51	100	100	0
	Child <=36mo	85.3	2343	Ref					
	Child >36mo	84.1	2527	0.91					
16^a^	**Province**				**< 0.0001**	0.69	100	100	0
	Province 1	87.9	806	Ref					
	Province 2	70.0	730	0.32					
	Province 3	81.1	1163	0.59					
	Province 4	87.4	495	0.96					
	Province 5	91.4	616	1.46					
	Province 6	98.0	740	6.76					
	Province 7	96.0	320	3.31					
17^a^	**Ecological Belt**				**0.0002**	0.60	100	100	0
	Mountain	96.1	885	Ref					
	Hill	88.3	1923	0.31					
	Terai (Plain)	80	2062	0.16					
	**Multivariable Main Effects Model**	**84.7**	**4870**			**0.96**	**86.8**	**99.6**	**80.8**

Sections 1–17 of this table describe results from univariable models; only the final row of the table describes performance of the multivariable model

^a^Denotes a variable that was retained in the main effects model

Twelve variables with univariable *p*-values below 0.25 met the criteria to remain in the initial multivariable model and in a final round of variable checking, one of the original variables with univariable p-value > 0.25 (child age group) entered the main effects model with a p-value smaller than 0.05, so was retained. Caregiver marital status and caregiver religion had p-values below 0.05 but were dropped because a) marital status led to a very large odds ratio for the *N* = 33 unmarried women and b) religion appeared to be colinear with ethnic group and including both in the model yielded an extremely high odds ratio for the Muslim ethnic group. Dropping these two covariates did not notably change the prediction performance of the model. The variables in the main effects model are listed in Table 5 and the classification performance of the main effects model is described in the final row of Table 4. The odds ratios and model output of the main effects model are shown in Table 6. The main effects model yielded a c-statistic of 0.96, which is considered “*outstanding discrimination”*. Note in Table 4 that the prediction performance of the main effects model is very nearly equivalent to that of the univariable model with ‘caregiver knew about campaign’. An electronic supplement includes additional commentary on the univariable and multivariable regression models.

**Table 5:** Variables in the main effects model

1. Program intervention: Caregiver knew about Campaign	
2. Geographic area or setting:	
2.1. Urban/rural Cluster	
2.2. Province of residence	
2.3. Ecological Belt	
3. Caregiver individual characteristics:	
3.1. Highest level of caregiver Education (3 groups) - Did not disclose or less than Primary, Primary, More than Primary	
4. Ethno-religious group characteristic:	
4.1. Ethnic Group	
5. Family characteristics:	
5.1. Source of Income	
5.2. Someone in family works abroad	
5.3. Number of Family Members in household	
5.4. Number of Children 9–59-month-old in household	
6. Individual child characteristic: Child Age Group [This variable was not originally in the multivariate model. But after testing its significance with the preliminary main effects model, was found to be significant and thus entered the final main effects model.]

**Table 6:**
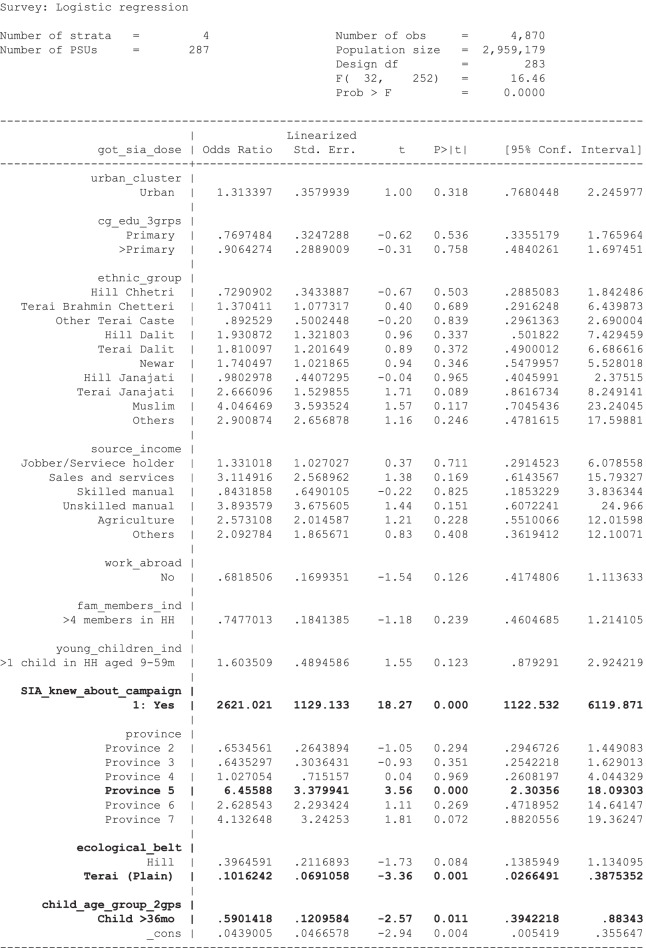
Logistic regression parameters for multivariable main effects model

_cons estimates baseline odds

Covariate levels with a *p*-value smaller than 0.05 are listed in bold font

## Discussion

The Gorkha earthquake in April 2015 and a major aftershock in May devastated Nepal and killed nearly 9000 people and injured 22,000 [19]. Measles outbreaks often follow natural disasters like these [20, 21]. The national immunization programme of Nepal responded promptly with a preventive SIA with measles-rubella and oral polio vaccines within 4 months of the earthquake in the affected districts (1st phase). The remaining districts were covered through a phased (three phases) campaign between February and April 2016. The campaign was effective in preventing any major outbreak of measles or rubella. In 2015, 2016 and 2017 there were 272, 282 and 99 confirmed measles cases and 9, 22 and 21 confirmed cases of rubella [22].

This post-campaign coverage survey (PCCS) estimated that the SIA campaign vaccinated 83% of the target children in the immediate post-earthquake phase and achieved an overall coverage of 85% for all phases combined, ranging from 78 to 98% between the four phases. Thus the overall coverage was lower than the target of at least 90% coverage but coupled with a sustained relatively high coverage with at least one dose of measles containing vaccine in routine immunization, it was probably sufficient to prevent the anticipated post-disaster flare-up of measles transmission evidenced by the declining trend in cases seen in national surveillance data [5, 23]. The fourth phase in mountain districts achieved the highest coverage of 98%, whereas the third phase covering mostly Terai and hill districts had the lowest coverage at 78%. We did not specifically look at province level factors determining immunization status. However, immunization coverage in Terai districts, especially province 2 has been historically low and may require further investigation. Published coverage evaluation data from SIA in Nepal is scanty. An unpublished report from an SIA campaign with MR vaccine in 2013 estimated the national vaccination coverage was 89.3% in 9–59 month old children (WHO-IPD data). No disaggregation by terrain was available [24].

Other surveys have also shown lower coverage in routine immunization in some Terai districts or in provinces which comprise mostly of Terai districts [5, 25]. As these districts have lesser challenges with physical access as compared to mountain districts, the reasons for lower immunization coverage should be explored by examining societal, communication or other programmatic challenges rather than physical access challenges alone. Results from the survey were compared with administrative coverage. While the ranking of performance of each phase is somewhat maintained, i.e., higher coverage in phases 4 and 2 and lower in phases 1 and 3, survey estimates are lower. This can be explained by several factors including difficulties in exactly ascertaining age, vaccination in campaigns of children above and below the target age, and a mismatch (often difficult to quantify) between where a person gets vaccinated and where he/she resides, the latter more frequent in urban settings [26].

In our survey, a limited number of socio-economic and geographic characteristics were explored and none was found to be significantly associated with SIA immunization status except residence by ecological zone where the mountain districts had the highest coverage of 96.1% (95% CI: 89.3, 98.6) while the Terai districts had lowest coverage of 80.0% (74.4, 84.7). Other authors have recently explored inequalities in immunization and other maternal and child health interventions in Nepal noting that the equity gap has been rapidly narrowing [27].

Other correlates of immunization coverage were explored to obtain actionable information from this survey which could inform future immunization programme interventions. Having information about the campaign ahead of time was almost perfectly predictive of whether the child received the MR dose. A child whose caregiver knew about the campaign beforehand was 53.6 times more likely to be vaccinated than one whose caregiver did not.

It is simple and reasonably accurate to say that although some other factors are correlated with receiving the campaign dose, the strongest predictor is one that agrees with what we know about Nepali culture and thankfully, one which the Ministry of Health and Population has power to affect: *Nepali caregivers utilize child health services that they know about*. The strong recommendation that comes through in these analyses is to let caregivers know about the time and place of vaccination campaigns and most will avail themselves of the service. Reaching out to caregivers with information about SIA through interpersonal communication is a program intervention that can be implemented by program managers with the right strategy and resources. Prior communication before an SIA to increase awareness in the community has also been effective in other countries. In Haiti, a similar analysis showed that children from household with prior knowledge of the SIA were significantly more likely to get vaccinated in the SIA [28].

The survey had several limitations. The fieldwork was conducted more than a year after completion of the SIA campaign and because there was no child-level written record of the SIA doses given, there could have been recall bias in ascertainment of SIA vaccination status. However, in Nepal routine immunization is delivered on the same date of the month from fixed posts. Immunization campaigns with injectable vaccines are delivered from the same posts but on a different date and the target age groups are different which would help distinct recall of the SIA campaign. As FCHV and health workers are personally known to the caregivers in most villages through frequent interactions, it is unlikely that the history about prior information would be subject to substantial recall bias. The response rate in the survey was very high at 99.8% which would mitigate against biases from selection or non-response, and indirectly reflects the Nepali community’s openness and trust in sharing immunization related information.

The observed design effect in this survey was 8.6, which is over three times the value used when calculating the survey sample size (2.5). The observed intra-cluster correlation coefficient in this survey was 0.178 which is only slightly higher than the assumed value of 0.167. But the average number of respondents per cluster was 17 instead of the 10 that were targeted. Also, there was some heterogeneity in the survey weights which contributed to the design effect – a topic that was not addressed in WHO’s 2015 draft reference manual but was added to Annex B1 of the finalized manual in 2018 [7]. Some expected heterogeneity of weights should be taken into account in future immunization survey sample size calculations in Nepal. The number of target households per cluster may be reduced somewhat compared with the planning parameters used for this survey to avoid collecting data from more respondents per cluster than planned.

The regression analyses ignore supply-side covariates, using only the fixed effect of province to control for differences in how the campaign was advertised, supplied, and conducted. It would be a good idea for vaccination campaigns to closely document supply-side problems and furnish those records to coverage survey analysts, but in practice this is not usually prioritized. As mentioned by a helpful reviewer, it would be nice to also capture a rich set of covariates from several levels of the health system and use multilevel regression to explore factors that correlate with differences in campaign outcomes across subnational strata. Instead, the regression analyses reported here are limited to individual level covariates collected from children’s caregivers and therefore only useful for describing differences at the individual level and not aggregated to subnational strata.

A final limitation is that a cross-sectional survey cannot be used to confirm causal relationships between respondent characteristics and whether they received the campaign dose. The regression analyses reported here describe correlation, but not necessarily causation.

This is the first time that the current WHO-vaccination cluster survey guidance was applied in Nepal. Beyond navigating difficult terrain in some of the selected clusters, there were no serious operational issues faced during selection of the primary sampling units or in the fieldwork to select households and caregivers. This showed that the new guidance based on a fixed number of households per cluster, rather than the earlier 30 × 7, or any quota sampling method for children used for immunization cluster surveys, is feasible to apply even in developing countries with a diverse terrain like Nepal [7].

Nepal has recently conducted another nationwide MR SIA campaign in 2020. Although the detailed analysis presented here was not yet known at that time when this SIA was being initially planned in 2018, for conduction in 2019, preliminary data from this PCCS had indicated that reaching prior information to caregivers would be a key intervention to increase coverage and that information received through interpersonal interaction with FCHV and local health worker was the most important source of prior information. The 2020 MR SIA campaign thus strategically planned to distribute invitation cards to caregivers through house visits by FCHV and health workers before the campaign, as well as SIA cards to caregivers as a record of SIA vaccination and undertook other mass communication strategies described below. Our analyses vindicate those strategic decisions.

For those caregivers whose children were not vaccinated in the 2015–2016 MR campaign, 93% mentioned lack of information as the main reason for not getting vaccinated. Only about 20% also mentioned any fear of adverse events following immunization as the reason for not getting vaccinated. As Nepal rolls out COVID-19 vaccination programme in 2021, these results should inform decisions regarding interpersonal communications through FCHV or local HW to harness community demand for COVID-19 vaccination.

It was well understood that communication would hold the key for successful immunization campaigns in Nepal. In the 2020 campaign, within the framework of vaccination as a human right as envisaged in the Immunization Act of Nepal, mayors and municipal chairpersons, non-governmental organization and local volunteers (FCHV, mothers’ groups and school personnel) were involved to make it a success [29].

This study explored for evidence of factors influencing positive deviance behaviour in supplementary immunization activities. In Nepal the programme intervention of giving prior intimation to caregivers through interpersonal communication worked with such astounding impact presumably because other components of the immunization value chain (micro-planning, cold chain and logistics, timely sessions, safe injections, management of adverse events etc.) also functioned properly. Using survey data to identify one or more specific actionable programme intervention(s), like better communication in this case, which influence positive deviance behaviour, can be pursued by immunization programmes and considered as part of a global best practice package.

## Supplementary Information

**Additional file 1: Supplement 1-Nepal PCCS.** Additional detail and commentary on logistic regression models.

## Data Availability

The datasets used and/or analysed during the current study available from the corresponding author on reasonable request. Also, the link to .zip file holding the anonymized vaccine coverage quality indicator (VCQI) tool compatible datasets, VCQI control program, VCQI augmented dataset, codebooks, Stata .do-file that estimates the logistic regressions from Table 4 and makes the figure in supplement #1, and a link to a .zip file holding the VCQI source code used for the analysis. https://www.dropbox.com/s/el6qcqyeyrj05un/Nepal%202016%20PCCS%20measles%20data%20and%20analysis%20programs.zip?dl=0

## Footnotes

1 More details about the SIA implementation can be found in the SIA report, which is available upon request to the authors.
