# Supplementary material for: Who gets vaccinated in a measles-rubella campaign in Nepal?: results from a post-campaign coverage survey
Source: BMC Public Health. 2022 Feb 3;22:221. doi: 10.1186/s12889-021-12475-0 (PMC8812357; doi:10.1186/s12889-021-12475-0)
Supplement: Supplementary file 1 — Additional file 1: Supplement 1-Nepal PCCS. Additional detail and commentary on logistic regression models. [file 12889_2021_12475_MOESM1_ESM.docx]

**Supplement 1-Nepal PCCS. Manuscript title “Who gets vaccinated in a measles-rubella campaign in Nepal?: results from a post-campaign coverage survey”**

**Additional Commentary on the Logistic Regression Models**

A logistic regression model estimates coefficients that may be used to calculate the probability that a respondent with a particular pattern of demographic variables was vaccinated in the campaign. A perfect regression model would assign a high probability of the vaccination outcome to those who were vaccinated, and a low probability to those who were not vaccinated.  Univariable logistic regression is conceptually simple because the probability of vaccination that it assigns is exactly equal to the weighted proportion of children in the sub-group who were vaccinated.  If the explanatory variable is child sex and 85% of boys were vaccinated and 82% of girls were vaccinated, the regression will assign every boy 85% probability of vaccination and will assign every girl 82% probability of vaccination.

For prediction purposes, one informative threshold is whether the model assigns a probability ≥ 50%, in which case we might informally say that the model predicts that the child would be vaccinated, or whether it assigns a probability < 50%, where we might informally say the model predicts the child is not likely to be vaccinated.  One does not strictly need to select 50% as the threshold for predicting the outcome, but 50% is a very logical threshold for assessing model performance.

In this survey, while some of the other univariable predictors show some power to discriminate between children who were vaccinated and those who were not, Table 4 indicates that models except ‘Knew about campaign’ predict > 50% probability of vaccination for every child in the dataset. These predictions are not particularly useful. This is precisely because, as indicated in Table 4, the majority of children in every subgroup defined by these other 16 variables were vaccinated. None of the other univariable models approach the excellent metrics of the model containing whether the caregiver knew about the campaign before it happened.

We note again here that Table 4 indicates the univariable model that considers ‘knew about campaign’ had very similar prediction performance metrics as the main effects model with that variable and ten others. Figure S.1 shows respondent level predicted probabilities for those two models. The left panel shows predictions for all those who were not vaccinated. The right panel show those who were vaccinated. In both panels, predicted probabilities for the univariable model are shown with gray o’s (stacked at p=1.8% and p=96.5% depending on whether the caregiver had heard of the campaign). Those whose univariable probabilities were 96.5% have their main effects model probabilities plotted using red x’s. Those whose univariable probabilities were 1.8% have their main effects model probabilities shown in blue. If the univariable model predicted perfectly, all the gray o’s in the left panel would fall below 50% and all the gray o’s in the right panel would fall above 50%. But some respondents who were not vaccinated heard about the campaign, so their predicted probability is high. And a few respondents who were vaccinated reported that they did not know about the campaign ahead of time. (They must have been in the right place at the right time to have their child vaccinated.) So the univariable model assigns them a low probability of being vaccinated.

Figure S.1: Predicted Probabilities from Main Effects Model and Best Univariable Model


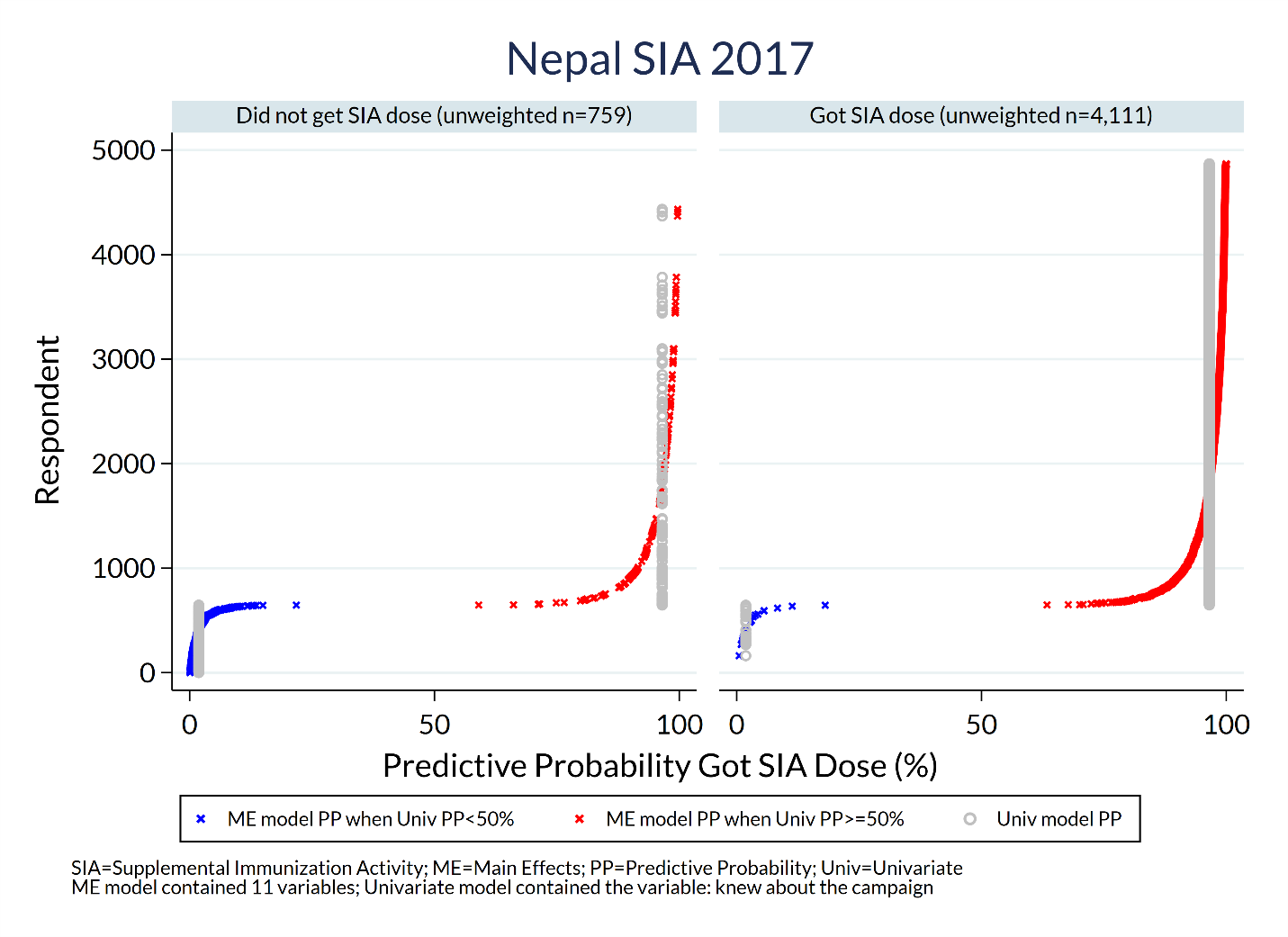


Note that although the red x’s spread the predicted probabilities of those who heard about the campaign above and below 96.5%, none of them are pushed down below 50%. Similarly, the blue x’s spread the predicted probabilities of those who did not hear about the campaign around 1.8%, but no blue x falls above 50%. Adding ten additional statistically significant covariates to the main effects model does not move the predicted probability for any respondent across the conceptually important 50% line. In a simple sense, both models predict the same qualitative outcome for every respondent and in that sense, the extremely parsimonious univariable model captures the important aspects of the more complicated main effects model.

To be sure, the model could probably be complicated further with some two- or even three-way interactions that would push red x’s below 50% or push blue x’s above 50%, and those models might predict some outcomes better than the excellent univariable model. But taken together, the models explored in the manuscript tell a simple story: The vast majority of caregivers who heard about the campaign took their children to be vaccinated. This quantitatively robust insight is simple, it correlates with what is known by Nepali immunization authorities, and it is very actionable.

**Goodness of fit**

It is recommended to report goodness of fit for logistic regression models (Hosmer & Lemeshow & Sturdivant, 2013) and there is a user-contributed Stata command that adapts Hosmer & Lemeshow’s goodness of fit statistic to be used with data from survey samples. The command compares the number of observed vs. predicted positive outcomes in weighted deciles of risk in the survey-adjusted regression dataset. (Archer & Lemeshow, 2006). Assessing goodness of fit on the main effects model reported in the manuscript rejects the null hypothesis of good fit with a p-value of 0.030. This seems to indicate that the model does not fit the data well. We note, however, that there are some substantial outliers in the survey weights for this dataset. Outliers can strongly affect the goodness of fit. Figure S.2 is a histogram of the post-stratified weights with vertical lines marking the 50^th^, 95^th^, and 99^th^ percentiles.

It may be instructive to trim the highest weights – to reduce their values to equal a threshold that we use to define outliers. Different analysts might select different criteria for defining outliers and might therefore trim the weights of more or fewer respondents. Defining outliers as those whose weight exceeds 3.5 times the median weight is one strategy discussed in Valliant, Dever & Kreuter (2013). In this dataset, 1,745 respondents in 109 clusters have weights higher than 3.5 times the median weight. More modest definitions might identify outliers as those whose weight exceeds either the 95^th^ or 99^th^ percentiles.

Trimming the survey weights to 3.5x the median, or to the 95^th^ percentile, or to the 99^th^ percentile affects N= 1,745, N= 228, or N= 39 respondents, respectively, in N= 109, N= 15, or N= 2 clusters and yields goodness of fit p-values of 0.786, 0.092, and 0.086, any of which would fail to reject the null hypothesis of good fit. We compared the model coefficients and discrimination characteristics for all three trimming possibilities with those of model fit with untrimmed weights and found that none of the three trimming thresholds affect the model’s discrimination performance or affect which model covariates and levels yield p-values smaller than 0.05. In summary, the apparent lack of fit in the main effects model is due to a small number of outliers in the post-stratified weights. These represent clusters that had very small probability of selection initially and whose weights were further inflated in the process of post-stratifying so the sum of weights in each province would be proportional to the population who were eligible for the measles campaign dose. Even modest trimming of the weights yields acceptable goodness of fit and neither modest nor aggressive trimming affects any conclusions drawn from the model. These sensitivity analyses may be reproduced using the datasets and Stata syntax provided in the ZIP file available at <https://www.dropbox.com/s/el6qcqyeyrj05un/Nepal%202016%20PCCS%20measles%20data%20and%20analysis%20programs.zip?dl=0>.

Figure S.2: Histogram of Post-stratified Survey Weights


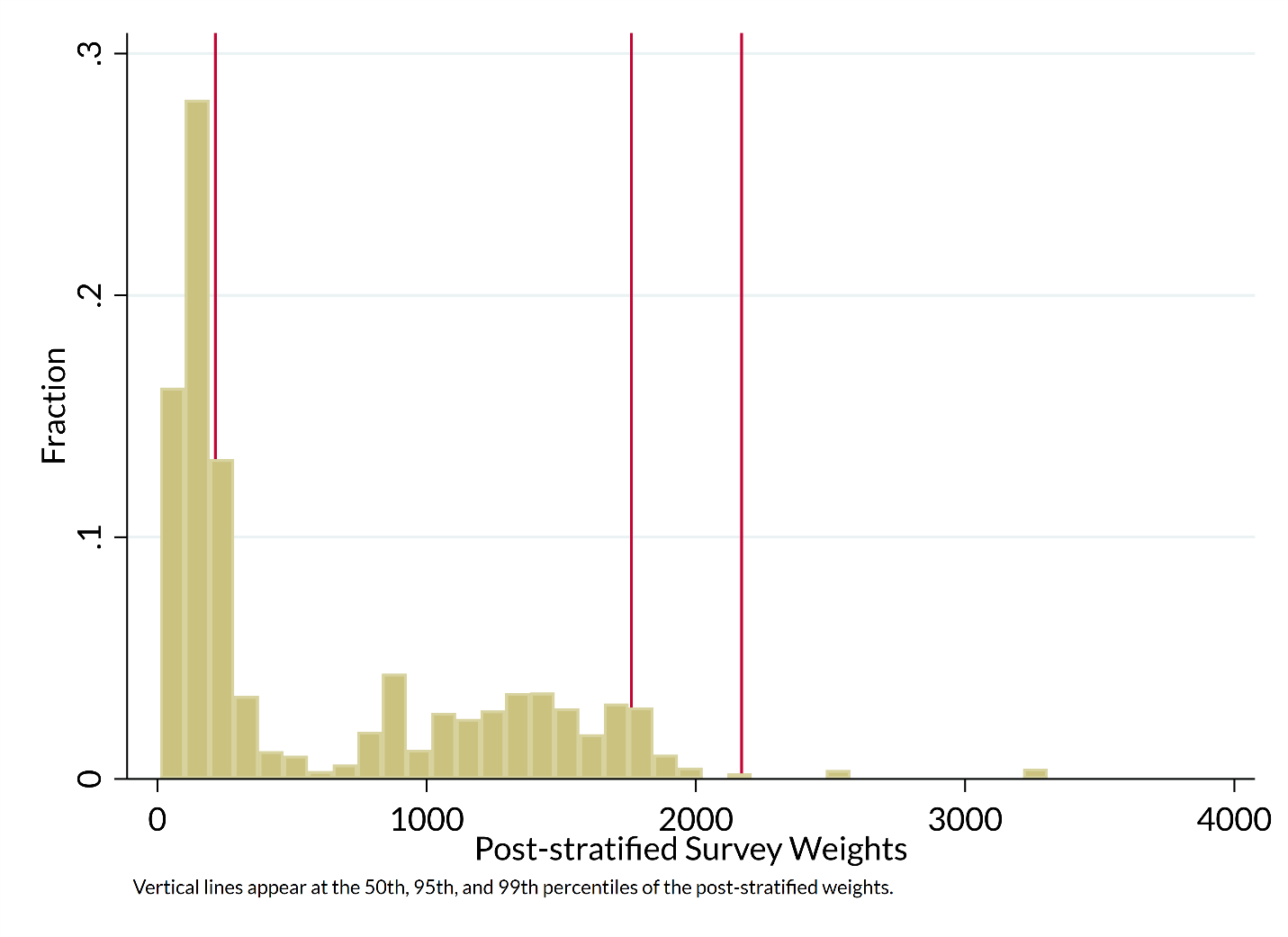


**References**

Archer, Kellie J., Stanley Lemeshow, and others. 2006. “Goodness-of-Fit Test for a Logistic Regression Model Fitted Using Survey Sample Data.” *Stata Journal* 6 (1): 97–105.

Hosmer, David W., Stanley Lemeshow, and Rodney X. Sturdivant. 2013. *Applied Logistic Regression, 3rd Edition*. 3rd ed. Hoboken, New Jersey: John Wiley & Sons, Inc. <https://www.wiley.com/en-us/Applied+Logistic+Regression,+3rd+Edition-p-9780470582473>.

Valliant, Richard, Jill A. Dever, and Frauke Kreuter. 2013. *Practical Tools for Designing and Weighting Survey Samples*. New York, NY: Springer New York. <http://link.springer.com/10.1007/978-1-4614-6449-5>.
